# Destructive Leadership and Turnover Intention among Chinese Rural Kindergarten Teachers: The Mediation of Ego Depletion and the Moderation of Kindergarten Affiliation

**DOI:** 10.3390/ijerph20032030

**Published:** 2023-01-22

**Authors:** Can He, Jie Xiong, Yue Zhang, Haiyuan Dou, Jiahui Du

**Affiliations:** 1 College of Education Science, Hubei Normal University, Huangshi 435002, China; 2 School of Education, Central China Normal University, Wuhan 430079, China; 3 Center for Mental Health, Wuhan University of Technology, Wuhan 430070, China

**Keywords:** destructive leadership, turnover intention, ego depletion, kindergarten affiliation, rural kindergarten teachers

## Abstract

One of the main challenges to the growth of early childhood education in rural China is the high teacher turnover rates. This study investigated the association between destructive leadership and turnover intention, as well as the mediating function of ego depletion and the moderating role of kindergarten affiliation, based on social exchange theory and ego depletion theory. A total of 409 Chinese rural kindergarten teachers were selected to complete a questionnaire on destructive leadership, ego depletion, and turnover intention. The results revealed that destructive leadership, ego depletion, and turnover intention were positively correlated. After controlling for age, destructive leadership was a positive predictor of turnover intention. The mediation model test revealed that ego depletion acted as a mediator between destructive leadership and turnover intention. Moreover, kindergarten affiliation mitigated the impact of destructive leadership on ego depletion. This effect is more pronounced in public kindergarten teachers compared to private kindergarten teachers. This study adds to our knowledge of the contributing factors and functioning mechanisms underlining turnover intentions among rural kindergarten teachers. It also provides new perspectives for policymakers and administrators to address rural kindergarten teacher attrition.

## 1. Introduction

A skilled, professional, and stable teaching staff is one of the key drivers for high-quality early childhood education (ECE). Despite the importance of these teachers, their high turnover rate has become a global concern [1,2,3]. For example, the turnover rates of teaching staff range from 15 to 30% in the United States [4], and in some Australian childcare institutions, this rate reaches 60% [5]. As one of the largest developing countries in the world, China is facing the same dilemma with an underdeveloped ECE system. A survey reported that one-third of kindergarten teachers resign each year in Beijing [6], and the circumstance in rural kindergartens is far worse [7]. The scarcity of rural kindergarten teachers, which has reached 640,000 [8], is made worse by the high turnover rates. Only 16.9% of rural kindergartens reached the minimal national standard for the teacher-to-child ratio, according to a survey conducted across 12 Chinese provinces [9]. Since the high turnover rates negatively affect the ECE and pose a huge challenge to the kindergarten teaching force [10], it is worthwhile to thoroughly investigate the contributing factors of turnover among rural kindergarten teachers.

Turnover intention is the strongest indicator of actual turnover behavior [11], defined as the inclination to quit [12]. Prior research has demonstrated that a variety of individual and environmental factors affect rural kindergarten teachers’ intention to leave their positions [13,14,15,16]. Leadership styles, which are significant environmental variables, have been shown to influence kindergarten teachers’ intention to quit. However, earlier studies have emphasized the effect of positive leadership styles [2,17] while ignoring the dark side of leadership, especially destructive leadership. Destructive leadership refers to volitional behaviors of the leader harming the organization and followers, which is a wide construct that incorporates behaviors in leadership [18]. A series of negative outcomes, such as decreased organizational commitment and increased turnover intention, can be triggered by destructive leadership [19], which has been a persistent problem for organizations. Fewer studies have been carried out on destructive leadership and its impacts in kindergarten settings, to our knowledge. China is a collectivist country influenced by the traditional Confucian culture, with a characteristic of large power distance. The relationship between leaders and subordinates is characterized by “superiority and inferiority”, resulting in the prevalence of destructive leadership in China [20]. Rural areas are an important breeding ground for traditional Chinese culture, and kindergartens in China are typical hierarchical organizations, making destructive leadership may be more evident in rural kindergartens. Further research is necessary to determine whether destructive leadership affects rural kindergarten teachers’ intention to resign.

Based on the aforementioned concerns, this study goes further to investigate the link between destructive leadership and turnover intention with rural kindergarten teachers as the research participants. To address rural kindergarten teacher attrition, the potential functioning mechanisms are also examined. Specifically, social exchange theory and ego depletion theory are used to investigate the association between destructive leadership and turnover intention, the mediating role of ego depletion, and the moderating effect of kindergarten affiliation.

### 1.1. Relationship between Destructive Leadership and Turnover Intention

Destructive leadership has become a prominent workplace stressor that negatively affects employees. Relevant studies have shown that destructive leadership can result in emotional exhaustion, negative emotions (such as depression and anxiety), reduced work performance and job satisfaction, turnover intention, and other negative consequences among subordinates [19,21,22]. The social exchange theory suggests that social interaction is an exchange activity in which individuals should follow the norm of reciprocity [23]. According to this theory, leaders and subordinates are in an exchange relationship based on the reciprocity norm. If the leader positively treats the subordinate, the subordinate will reciprocate with corresponding attitudes and contributions. On the contrary, if the leader treats the subordinate in a controlling, threatening, and manipulative manner, the subordinate will rarely respond in the same hostile manner due to the inequality of power [24]. This inequality can lead to the growing dissatisfaction of subordinates, causing them to quit their jobs as an indirect way of expressing their dissatisfaction. Related studies confirm that destructive leadership increases employee turnover intention [19,22]. Based on the above studies, H1 is proposed:

**H1.**  *Destructive leadership is positively related to rural kindergarten teachers’ turnover intention*.

### 1.2. Mediation Effect of Ego Depletion

The ego depletion theory states that the strength of self-control is finite and essential for the executive part of the self [25]. Since self-control behaviors employ the same resources, the resources can be easily depleted. After effortful acts of will, the self-control resources enter a state of diminishment and reduction, referred to as ego depletion [26,27,28]. According to the ego depletion theory and prior studies, ego depletion may act as a mediating factor between destructive leadership and turnover intention. Destructive leadership may deplete the psychological resources of an individual and trigger ego depletion. Confronted with negative behaviors such as control, threats, and ridicule from leaders, followers need to control primitive retaliatory impulses from interpersonal provocation [26,29] and cope with diminished self-esteem caused by “losing face” in front of colleagues [30]. During this process, the limited self-control resources of an individual are depleted, leading to ego depletion. At the same time, destructive leadership induces negative emotions in followers, such as tension, anger, and frustration [19,22]. Given the unequal status of both parties, they may have to suppress negative emotional expressions. This action can further drain self-control resources, weakening the power of self-control. In addition, studies have also confirmed that destructive leadership (abusive supervisor) can exacerbate ego depletion among followers [31].

Ego depletion may result in the intention to resign. According to the ego depletion theory, employees’ resources for self-control are depleted as a result of fulfilling work obligations and accomplishing work objectives. Ego depletion may lead to reduced self-regulation and cognitive biases, and employees may underestimate their capacity to manage the outside working environment, thus leading to work alienation [32]. Work alienation makes individuals reduce their social interactions and separate themselves from the work environment. Then, their desire to maintain stable relationships with colleagues and work hard for the organization decreases, resulting in a declined organizational commitment [33,34]. The decline in organizational commitment is a key precursor to turnover, according to the casual models of employee turnover [35,36]. Additionally, ego depletion has been demonstrated to increase the tendency to resign [37]. Under the influence of ego depletion, individuals become psychologically detached from their work and socially separated from other employees, which lowers their commitment to the organization and increases the likelihood of resigning. On this basis, H2 is proposed:

**H2.**  *Ego depletion may act as a mediator between destructive leadership and turnover intention*.

### 1.3. Moderation Effect of Kindergarten Affiliation

According to ego depletion theory, destructive leadership is indirectly associated with turnover intentions through ego depletion. Contextual factors are also crucial to take into account, as they may help to explain why some subordinates are more vulnerable to the ego-depleting effects of destructive leadership than others. Based on relevant theories and prior research, we propose that kindergarten affiliation may moderate the association between destructive leadership and ego depletion. By affiliations, Chinese kindergartens are broadly divided into public (owned by the government, army, public institutions, communities, and local state-owned enterprises) and private (owned by individuals or non-governmental departments). According to the statistics from the Ministry of Education of the People’s Republic of China [38], public kindergartens accounted for approximately 43% of Chinese ECE programs in 2021. In China, public kindergartens are commonly funded by educational authorities and other state entities [39]. In comparison to private kindergartens, they frequently have better resources, equipment, and salaries [40,41].

However, public kindergartens are often subject to more external restrictions and supervision. This highly centralized management mechanism results in low autonomy for teachers. On the contrary, the operation mechanism of private kindergartens is flexible. To improve the efficiency of kindergartens, private kindergartens tend to encourage voice and innovative behaviors. Therefore, teachers have higher autonomy and a greater voice in the curriculum and teaching [42,43]. The self-determination theory states that activities that hinder the need for autonomy deplete the energy of the individual, while activities that fulfill the fundamental psychological demand for autonomy boost vitality and help regain the strength of self-control [44]. Previous studies have shown that job autonomy moderates ego depletion [45,46,47,48], with “have to” types of activities being more ego-depleting than “want to” types of activities [49]. Job autonomy empowers employees by giving them more freedom and control, thus enabling them to work more autonomously and willingly [45]. According to the self-determination theory, private kindergarten teachers may have more resources for self-control to handle the psychological strain, cognitive dissonance, and negative emotions brought on by destructive leadership, which lessens its effect on ego depletion. When compared to teachers in public kindergartens, destructive leadership may have a less significant impact on ego depletion. Therefore, the H3 is proposed:

**H3.**  *The association between destructive leadership and ego depletion is moderated by kindergarten affiliation*.

**Figure 1:**
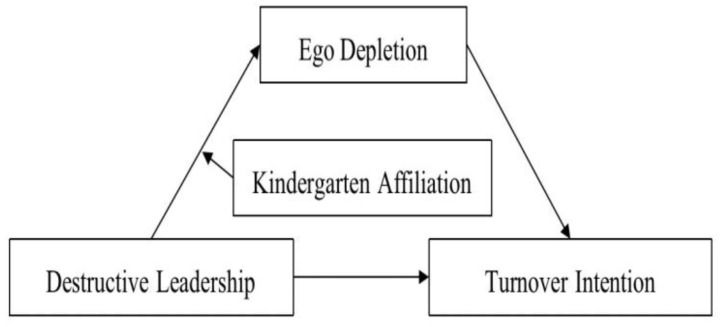
The proposed moderated mediation model.

To sum up, this study’s main objective was to further our understanding of how destructive leadership affects rural kindergarten teachers’ intentions to leave their jobs. First, we intended to investigate the association between destructive leadership and turnover intention. Second, by investigating the mediating function of ego depletion and the moderating effect of kindergarten affiliation, we aimed to uncover the functioning mechanisms underlying the relationship. We anticipate that findings from this study will be applied to provide new perspectives for policymakers and administrators to address rural kindergarten teacher attrition. Figure 1 displays the hypothetical model.

## 2. Materials and Methods

### 2.1. Participants

The participants were 409 teachers recruited from rural kindergartens in Hubei Province in China. All participants were full-time and female teachers. The sample’s average age was 32.94 years old (SD = 7.05) and average work seniority was 5.16 years (SD = 4.13). Of the participants, 174 came from private kindergartens and 235 from public kindergartens. The sample’s sociodemographic details are outlined in Table 1.

### 2.2. Procedure

We obtained permission from Hubei Normal University’s Ethics Committee for Scientific Research before carrying out the survey. In the current study, convenience sampling has been adopted. Given the need for prevention and control of the COVID-19, data were collected through an online questionnaire platform. Moreover, questionnaires were distributed with the help of the Huangshi Municipal Bureau of Education. Before the investigation, participants’ informed consent was obtained. All the participants were required to independently, truthfully, and anonymously complete the questionnaires. A total of 440 rural kindergarten teachers in Hubei Province were recruited in October 2021 for the investigation. About 409 valid questionnaires were collected after removing those with missing information and those that finished too quickly (under three minutes).

### 2.3. Measures

#### 2.3.1. Destructive Leadership

Destructive leadership was measured with a five-item scale developed by Mitchell and Ambrose [50], which has been revised and used in the Chinese cultural context [51]. A sample item was “Ridicules subordinates”, with responses rated on a scale of “1 = strongly disagree” to “6 = strongly agree”. Higher scores reflect more destructive leadership. In this study, Cronbach’s α for this scale was 0.95.

#### 2.3.2. Ego Depletion

A five-item scale designed by Twenge, Muraven, and Tice [52] was used to measure ego depletion. It has been successfully used in the study of Chinese cultural background [53]. On a four-point Likert scale, participants were asked to rate the degree to which each item accurately captured how they felt. Higher scores indicate more ego depletion. In the current study, Cronbach’s α was 0.86.

#### 2.3.3. Turnover Intention

A three-item scale created by Konovsk and Cropanzano was used to measure the turnover intention [54], which was equally applicable to the Chinese samples [55]. A sample item was “How often do you think about quitting your job at this organization” with responses rating on a scale of “1 = very unlikely” to “5 = very likely”. A higher score reflects a greater inclination to resign. Cronbach’s α for this scale was 0.91.

### 2.4. Data Analyses

The data analyses were carried out using SPSS 25.0 (IBM, Armonk, NY, USA) and Mplus 8.3 in the following steps. First, descriptive statistics (i.e., means and standard deviations) were calculated for all variables, and for psychometric scales, Cronbach’s α reliability was estimated. Second, common method variance was tested by the structural equation model. Third, with two-tailed significance testing, Pearson’s correlation coefficients were computed to perform correlation analysis. All statistical tests were conducted with a significance level of *p* < 0.05. Finally, the moderated mediating model was tested with SPSS macro program PROCESS [56]. Specifically, the mediation of ego depletion on the effect of destructive leadership on turnover intention was tested using Model 4 in the PROCESS template, while the moderating role of kindergarten affiliation on the mediated effect was tested using Model 7 in the template. Given that PROCESS Marco does not provide standardized regression coefficients, z-scores of all continuous variables were generated before performing regression analyses. In addition, a dummy variable was created to represent kindergarten affiliation, with private kindergartens being coded as 0 and public kindergartens being coded as 1. During the processing of the PROCESS Macro, 5000 bootstrap samples were generated to approximate the confidence interval (CI) of the indirect effect based on the original sample, with no zero 95% CI indicating statistical significance. Additionally, age was controlled in all regression models.

## 3. Results

### 3.1. Common Method Deviation Test

In the present study, to test for common method variance, confirmatory factor analysis was carried out using the structural equation model [57]. Using Mplus 8.3, a one-factor model was examined, and the fit indices indicated a poor fitting of the model to the data (χ^2^/df = 28.15, RMSEA =0.26, CFI = 0.66, TLI = 0.60), indicating no significant common method bias.

### 3.2. Preliminary Analyses

Table 2 displays the descriptive statistics of all variables. As shown in Table 1, destructive leadership had a positive correlation with turnover intention (r = 0.50, *p* < 0.01) and ego depletion (r = 0.40, *p* < 0.01). Ego depletion and turnover intention had a positive correlation (r = 0.55, *p* < 0.01).

### 3.3. Testing for the Mediating Role of Ego Depletion

Model 4 in the SPSS macro-PROCESS was employed to investigate the potential link between destructive leadership and turnover intention, and also the possible mediating role of ego depletion. According to Table 3, destructive leadership positively predicted turnover intention (β = 0.51, *p* < 0.01); destructive leadership positively predicted ego depletion (β = 0.40, *p* < 0.01); ego depletion positively predicted turnover intention (β = 0.41, *p* < 0.01). Finally, destructive leadership strongly predicted turnover intention through ego depletion, as shown by the bias-corrected bootstrap mediation test, indirect effect = 0.17, boot SE = 0.03, 95% CI = [0.11, 0.22]. The association between destructive leadership and turnover intention was partially mediated by ego depletion. In addition, the mediation effect contributed 33.33% of the total effect. Therefore, H1 and H2 can be supported by the mediation analysis results.

### 3.4. Testing for Moderated Mediation

**Figure 2:**
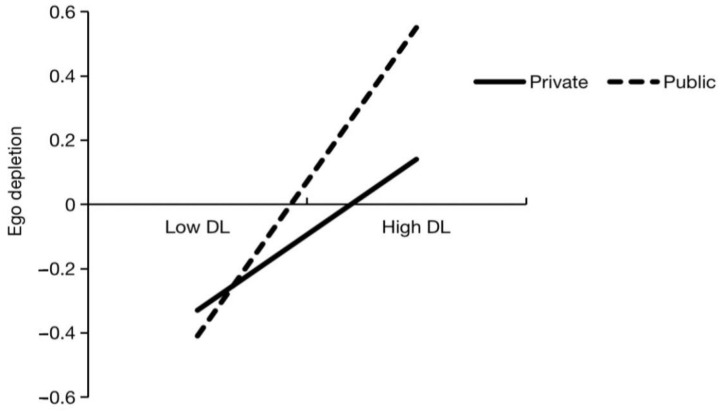
Interaction between DL and KS on ego depletion.

Model 7 in the SPSS macro-PROCESS was adopted to determine if kindergarten affiliation affected the correlation between destructive leadership and ego depletion. Table 4 presents the outcomes of the moderation analysis. The regression model showed a correlation between ego depletion and the interaction of destructive leadership and kindergarten affiliation (β = 0.25, *p* < 0.05). Simple slope tests revealed that destructive leadership significantly predicted ego depletion for private kindergarten teachers (B simple = 0.24, t = 3.06, *p* < 0.01, CI = [0.09, 0.39]), and the predictive effects were stronger for public kindergarten teachers (B simple = 0.49, t = 8.70, *p* < 0.01, CI = [0.38, 0.60]). The interaction diagram is depicted in Figure 2. The H3 is supported by these findings, which show that kindergarten affiliation moderates the indirect effects of destructive leadership on turnover intention.

## 4. Discussion

Based on social exchange theory and ego depletion theory, the association between destructive leadership and the intention to quit among Chinese rural kindergarten teachers was investigated. In addition, the mediation of ego depletion and the moderation of kindergarten affiliation were examined. The findings demonstrated a positive correlation between destructive leadership and rural kindergarten teachers’ inclination to quit, indicating that destructive leadership was a risk factor for turnover intention. Faced with destructive leadership, disadvantaged subordinates rarely respond in a hostile manner, but their dissatisfaction may accumulate over time, eventually leading to silent rebellion by leaving the organization. It is also worth noting that ego depletion acted as a mediator between destructive leadership and turnover intention. The turnover intention is formulated with increased ego depletion. Furthermore, kindergarten affiliation moderated the relationship between destructive leadership and ego depletion. Specifically, the impact of destructive leadership on ego depletion was greater for public kindergarten teachers than for private kindergarten teachers.

### 4.1. Theoretical Contributions

A number of theoretical implications can be drawn from this study. First, this study may represent a first step in exploring the connection between destructive leadership and turnover intention in kindergarten settings. It generalizes findings on the connection between destructive leadership and turnover intention from financial, retail, manufacturing, and service industries to the ECE field [22,58]. The adverse impact of destructive leadership on turnover intention was verified [18,21], and the research scope of destructive leadership was expanded. China has traditionally been a society that values “relationships” and “favors”. The leadership styles in Chinese organizations significantly affect the work attitudes and behaviors of subordinates. Previous studies also show that destructive leadership is more evident in the Chinese cultural context [51]. Based on these results, particular consideration should be given to the negative effects of destructive leadership, and strategies should be developed to address rural kindergarten teacher attrition from the perspective of leadership styles.

Second, this study explores how destructive leadership affects turnover intention and further confirms that ego depletion plays a mediating role in the harmful consequences of destructive leadership [37]. Prior research has focused on the impact of destructive leadership on the turnover intentions of subordinates in terms of stress, cognition, and emotion [58,59,60,61]. The current study, which is based on ego depletion theory, reveals the relationship between destructive leadership and turnover intention from the ego depletion perspective, advancing the understanding of the mechanisms underlying the aftereffects of destructive leadership. Although negative cognition, negative emotions, and stress can lead to turnover intentions, addressing negative cognitions, regulating negative emotions, and coping with stress can also deplete limited self-control resources [62], thus increasing their willingness to quit. Therefore, employing ego depletion as a mediator offers an integrative viewpoint for exploring the impacts of destructive leadership on turnover intention. Additionally, from the viewpoint of intervention, it is more practical to use ego depletion as the mediating variable. Addressing ego depletion is more effective than adjusting cognition and managing stress because it can be alleviated by rest and sleep in a short amount of time [25]. In summary, this study sheds light on the association between destructive leadership and turnover intentions in kindergarten settings from the ego depletion perspective. It also extends research on the consequential mechanisms of destructive leadership and broadens the applicability of ego depletion theory.

Third, to our knowledge, this study first demonstrates that kindergarten affiliation can moderate the influence of destructive leadership on ego depletion, further confirming that working environment characteristics can mitigate the harmful consequences of destructive leadership. Previous research has demonstrated that the consequences of destructive leadership differ depending on the characteristics of the workplace [31,37]. Kindergarten affiliation is a contextual factor that is objective as opposed to subjectively perceived workplace characteristics, such as organizational support and LMX differentiation [31,37]. For a long time, there has been an inequitable distribution of quality public resources between public and private kindergartens in China, with public kindergartens having more high-quality educational resources. On the contrary, many private kindergartens lack the financial support to ensure basic quality requirements [63]. Thus, the general public has higher expectations and recognition of management norms, teacher qualifications, and the teaching quality of public kindergartens [39]. Furthermore, most existing studies emphasize the advantages of public kindergartens, which has resulted in the potential problems of public kindergartens being ignored. Therefore, taking kindergarten affiliation as the moderator helps address the teacher attrition problem in rural kindergartens from a macro-management perspective of the government, suggesting that the drawbacks of public kindergartens should get consideration. In addition, research on the moderators of the ego depletion effect is furthered by this work. Prior research has mostly focused on the boundary conditions of ego depletion from the standpoint of subjective variables including personality traits, emotions, and cognition [64]. This study, to our knowledge, lays the groundwork for future research by offering the first insight into the impact of kindergarten affiliation on self-control resources in terms of objective variables.

### 4.2. Practical Implications

First, organizations should seek to reduce the potential for destructive leadership regarding its negative impact on ego depletion and intention to leave. For example, organizations should establish scientific selection mechanisms for managers. When selecting managers, candidates should be assessed in multiple aspects. Comprehensive factors (such as personal morality, personality characteristics, and interpersonal communication) should be incorporated into the evaluation criteria, thus preventing candidates with destructive leadership traits from entering kindergarten management. Furthermore, organizations should set up effective feedback channels and utilize third-party evaluation mechanisms to keep an eye on destructive leadership behaviors, which can help create a culture emphasizing leadership accountability, feedback, and employee participation. At the same time, leaders should be mindful of how they communicate with subordinates and encourage them to report the destructive behaviors of their superiors.

Second, our findings demonstrate the importance of preventing teachers’ ego depletion. Organizations should take effective measures to rescue the self-control resources of teachers. To combat the ego depletion effect, kindergartens can, for instance, create a low-pressure work environment through emotional communication and regular team-building activities that allow teachers to work in a happy mood [65]. Furthermore, given that the satisfaction of autonomy can mitigate the depletion of self-control resources [44], organizations should enable teachers to experience a sense of self-control in their work, protect their teaching autonomy, and meet their need for autonomy. Organizations can also train teachers regarding self-affirmation and self-control, which may effectively promote their self-control and assist them in quickly recovering from ego depletion [66]. To help teachers save and recharge resources, they should be encouraged to have short breaks at work.

Third, the empirical findings also indicate that education authorities should help to improve the management of public kindergartens. The education administration should relax the self-management authority and de-bureaucratization of rural public kindergartens. Excessive administrative power and intervention by higher-level authorities are striking problems in Chinese kindergarten management, especially prevalent in public kindergartens [67]. Under this bureaucratic management background, it is easy for kindergarten directors to adopt controlling and threatening management styles to execute orders from their superiors, resulting in destructive leadership in their work. Therefore, the education management department should simplify and decentralize the administration, change from a one-way leadership system based on supervision to a two-way interaction mechanism based on guidance and service, and delegate some management and decision-making powers to the principals. In addition, the professional training of principals of rural public kindergartens should be strengthened. In China, the principal responsibility system is implemented in kindergartens. Principals of public kindergartens are generally appointed by the local department of education, and most of them are directly transferred from elementary school teachers. Due to the lack of basic professional concepts and knowledge, they may often ignore the specificity and professionalism of ECE, resulting in negative leadership behaviors. On this basis, it is necessary to provide targeted training for public kindergarten principals, thus promoting the construction and professional development of rural kindergarten teachers.

## 5. Limitations and Further Research

Despite these findings and contributions, this study has several limitations. First, the cross-sectional study design that the conclusions are based on makes it challenging to establish causal interpretations. Future research should employ longitudinal designs to investigate causal links between the variables over long periods. Second, the present study adopted the self-report method, which may impact the authenticity of the results due to social desirability effects. In the future, multiple-source assessments can be performed to address this problem. Third, the convenience sampling of this study may result in sampling bias and lessen the reliability of the findings. Future research can make use of random sampling. Last, the present study only explored the moderating role of kindergarten affiliation. Other objective variables might also be susceptible to the impact of destructive leadership on the intention to quit. Future research can examine the possible boundary variables in terms of objective working contextual features, such as the scale of kindergartens and the degree of kindergarten quality. In this way, the relationship between disruptive leadership and turnover intention under different conditions can be further understood.

## 6. Conclusions

In summary, by developing a moderated mediation model, this study advances our knowledge of why and how destructive leadership is linked to Chinese rural kindergarten teachers’ intention to quit. Specifically, destructive leadership is found to be a risk factor for increased turnover intention. Furthermore, destructive leadership raises the probability of turnover intention through the mediation of ego depletion, and the mediating model is moderated by kindergarten affiliation.

## Figures and Tables

**Table 1:** Participant sociodemographic characteristics.

Categories		Frequency	Percentage (%)
Age (years)	≤20	11	2.7
	21–30	147	35.9
	31–40	199	48.7
	≥41	52	12.7
Work Seniority (years)	≤3	170	41.6
	4–5	94	23.0
	6–10	110	26.9
	11–15	29	7.1
	≥16	6	1.4
Education Level	High school, technical school, and below	227	55.5
	College degree	156	38.1
	Bachelor’s degree and above	26	6.4
Monthly Income (RMB, yuan)	≤1500	18	4.4
	1501–2500	313	76.5
	2501–3500	69	16.9
	≥3501	9	2.2
Kindergarten Affiliation	Private kindergartens	174	42.5
	Public kindergartens	235	57.5

**Table 2:** Means, standard deviations, and correlation coefficients for the main variables.

Variable	M	SD	1	2	3	4
1. destructive Leadership	2.12	0.95	—			
2. Ego Depletion	1.90	0.56	0.40 **	—		
3. Kindergarten Affiliation	0.57	0.50	0.03	0.09	—	
4. Turnover Intention	2.45	0.85	0.50 **	0.55 **	−0.12 *	—

Note: M and SD are abbreviations for the mean and standard deviation, respectively; ***N*** = 409, * *p* < 0.05, ** *p* < 0.01.

**Table 3:** Mediation analyses.

	Model (1) (Turnover Intention)			Model (2) (Ego Depletion)			Model (3) (Turnover Intention)		
	β	SE	t	β	SE	t	β	SE	t
Age	0.01	0.01	0.03	−0.01	0.01	−1.53	0.01	0.01	0.77
DL	0.51	0.04	11.72 **	0.40	0.05	8.81 **	0.34	0.04	8.01 **
Ego Depletion							0.41	0.04	9.77 **
R^2^	0.25			0.17			0.40		
F	68.67 **			40.33 **			88.26 **		

Note: DL = destructive leadership; ** *p* < 0.01.

**Table 4:** Moderated mediating analyses.

	Model (1) (Ego Depletion)			Model (2) (Turnover Intention)		
	β	SE	t	β	SE	t
Age	−0.01	0.01	−1.54	0.00	0.01	0.77
DL	0.24	0.08	3.06 **	0.34	0.04	8.01 **
KS	0.17	0.09	1.72			
DL × KS	0.25	0.10	2.61 *			
Ego Depletion				0.41	0.04	9.77 **
R^2^	0.19			0.40		
F	23.03 **			88.26 **		

Note: DL = destructive leadership; KS = kindergarten affiliation; * *p* < 0.05, ** *p* < 0.01.

## Data Availability

To protect the participants’ privacy, the original data used for the analysis are not publicly available but from the corresponding author at a reasonable request.
